# Research progress of circRNA in malignant tumour metabolic reprogramming

**DOI:** 10.1080/15476286.2023.2247877

**Published:** 2023-08-20

**Authors:** Yikun Geng, Min Wang, Zhouying Wu, Jianchao Jia, Tingyu Yang, Lan Yu

**Affiliations:** a Graduate school, Inner Mongolia Medical University, Hohhot, China; b Clinical Medical Research Center, Inner Mongolian People’s Hospital, Hohhot, China; c Inner Mongolia Key Laboratory of Gene Regulation of The Metabolic Disease, Inner Mongolian People’s Hospital, Hohhot, China; d Inner Mongolia Academy of Medical Sciences, Inner Mongolian People’s Hospital, Hohhot, China

**Keywords:** CircRNA, cancer metabolic reprogramming, malignant tumour, microRNA (MiRNA), glycolysis

## Abstract

Cancer is a multi-factor systemic malignant disease, which has seriously threatened human health and created a heavy burden on the world economy. Metabolic reprogramming, one of the important signs of malignant tumours, provides necessary nutrition for tumorigenesis and cancer development; thus, it has recently become a research hot spot, even though the metabolic mechanism is quite intricate. Circular RNA (circRNA) affects cancer cell metabolism through various molecular mechanisms, playing an important role in promoting or suppressing cancer. Because of the structure characteristics, circRNA is quite stable, and can be utilized as biomarkers. In this review, we analysed and summarized the characteristics and biological functions of circRNA and comprehensively reviewed and discussed the important role of circRNA in cancer metabolic reprogramming. This review will provide new ideas for developing new anti-cancer therapeutic targets, mining cancer diagnostic and prognostic markers, and will provide guidance for other researchers to design circRNA-related experiments and develop anti-tumour drugs.

## 1. Introduction

Malignant tumours (cancer), are major public health issues that seriously threaten human life. In 2020, the number of individuals with cancer diagnosis in the world was reported as 19.3 million [1], with a significant upward trend. The malignant nature of cancers requires substantially higher than normal energy supply, which utilizes the Warburg Effect [2] (anaerobic glycolysis), allowing tumour cells to quickly adapt to the micro-environment and quickly proliferate. The increased demand for energy in cancer cells calls for changes in the metabolic pathway to promote cell proliferation and growth, called metabolic reprogramming. This provides cancer cells with the ability to resist external stimuli, acquiring new functional features. Glucose, lipid and amino acid metabolism are greatly changed in malignant tumours [3]. Tumour suppression method developed from energy metabolism is a difficult problem but has received substantial research interest in recent years.

CircRNA is the non-encoded RNA, with a closed ring-shaped structure, thus without a 5’ or 3’ end and PolyA tail. CircRNA has proved to be closely related to various cancers, with circRNAs participating in the energy metabolic reprogramming of malignant tumours. Through elucidation of the mechanism of circRNA affecting the metabolism of cancer, effective treatment strategies might be determined. Due to their stability, circRNAs may be used as biomarkers and have great potential application in the diagnosis, treatment and prognosis of cancer. At present, there are many publications describing circRNA in tumour diagnosis and treatment, but few discussing the relationship between circRNA and tumour energy metabolism. Therefore, this article summarizes the mechanism of circRNA in regulating tumour energy metabolism, which is of great significance for understanding the relationship between circRNA and cancer metabolic abnormalities and disease treatment.

## 2. Characteristics and biological functions of circRNA

### 2.1. Biogenesis and characteristics of circRNA

CircRNA is closed circular structure formed by reverse splicing between the 3’ and 5’ ends of precursor messenger RNA (pre-mRNA). The generating process of circRNA is called back-spliced circularization, which includes three main types of mode [4–6]: canonical spliceosomal machinery-dependent mode is the most common mechanism which occupying nearly 80%. The second mode is that cis-acting elements promote circRNA generating. The 3’ splice from the donor intron combines with the 5’ splice of the acceptor intron to form a loop structure, which is direct backsplicing driven by intron pairing. The third mode is that RNA binding proteins (RBPs) regulate the circRNA production.

Compared to linear RNAs, circRNAs cannot be degraded by Ribonuclease R (RNase R) due to the lack of 5’ and 3’ free terminals and can stably exist in various tissues [7]. In another words, circRNAs are stable in the environments out of the cells [8]. Besides, circRNA exists abundantly in exosomes. Tumor circRNAs are mainly distributed in the circulation system by packaging into exosomes [9]. Tumor cells can produce and release a large number of exosomes, and the amount is significantly higher than that from the normal cells [10]. Combined with the characteristics of stable circRNA structure and not easy to be degraded, exosomes can be used for clinical non-invasive or minimally invasive diagnosis, and can more efficiently monitor the molecular characteristics of cancer.

### 2.2. Physiological functions of circRNA

As is known, the classic-competing endogenous RNA (ceRNA) has been well researched in recent years. CircRNA acts as the miRNA sponge to adsorb miRNA blocking the binding of the miRNA to the target gene; thus, exerting a pro-tumour or tumour-suppressing effect in variety of cancers [11–13]. At present, a number of reports have revealed that circRNA plays an important role in regulating cancer gene expression, including the proliferation, invasion, migration and apoptosis of the cancer cells. For example, circAKT3 functions as a ceRNA to activate the PI3K/AKT signalling pathway in gastric cancer cells by sponge engulfing miR-198 and eliminating the inhibitory effect of this miRNA on its target gene PIK3R1 [13]. Specifically, miRNAs prevent translation of the target mRNA by complementary pairing with the 3’ UTR region of the target mRNA, thereby affecting the stability of the target mRNA.

Furthermore, circRNA competes for RBP to function, where RBP facilitates the reverse splicing process of circRNA [14]. RBPs can bind either double-stranded or single-stranded RNA, and some RBPs have complete RNA-binding domains that can bind to specific sequences [15]. CircRNA can regulate the occurrence and development of cancer by interacting with RBP [15]. For example, in hepatocellular carcinoma (HCC) cells, cLARS, a type of circRNA, down-regulated ALKBH5 which is a kind of RBP to inhibit tumour cell apoptosis [16]. On the other hand, RBP could affect the formation of circRNA, and different RBPs play either promoting or inhibiting roles in the process of circRNA back-splicing [17]. Previous reports have demonstrated that the Quaking (QKI) family of proteins play an important role in RNA looping with the insertion of synthetic QKI-binding sites into introns to generate circular RNA [18]. QKI5 was shown to regulate circZKSCAN1 formation, when QKI5 was upregulated in HCC cells, the expression of circZKSCAN1 was also significantly increased [14]; while ADAR1, another RBP, inhibits circRNA formation [19].

In eukaryotic cells, the 5 ‘and 3’ ends of RNA are essential for protein translation. Due to lack of 5 ‘and 3’ ends, circRNAs had not been considered as coding RNAs until recently increasing evidences have shown that certain circRNAs possess open reading frames (ORFs); suggesting that circRNAs have the potential to encode proteins [20]. In addition, circRNA may also translate into proteins and peptides in another unique way called ‘non-cap-dependent translation’. The initiation of internal ribosome entry sites and N6-methyladenosine-mediated translation is the important mechanism for circRNA translation [21].

CircRNA can also interact with RNA polymerase II to enhance the expression of its parent genes. Li reported that EIciRNA can bind to nuclear micro-ribonucleoprotein particles U1snRNPs to form elciRNA-U1 snRNPs complex, which can regulate the activity of RNA polymerase II to promote the transcription of parental genes [22]. Conn reported that circSEP3 could tightly bind to the homologous DNA site of its parental gene, thereby regulating the alternative splicing process [23].

## 3. CircRNA and cancer cell metabolic reprogramming

Metabolic reprogramming is one of the important features of cancer. With change of external nutrients and different stress conditions, cancer cells utilize different metabolic pathways to produce ATP and biological macromolecules for use. Aerobic glycolysis, increased glutamine decomposition, fatty acid biosynthesis and increased oxidative phosphorylation are included in such altered metabolism. Further, mitochondrial production capacity is affected, affecting the occurrence and development of malignant tumours. CircRNAs are involved in regulating metabolic reprogramming of various cancer cells, influencing energy uptake and utilization in cancer cells to either promote or inhibit the development of malignancies [24–27]. Further, circRNA regulates the growth and metastasis of malignant tumours by influencing substance transporters and key enzymes during energy metabolism via modulating transcription factors and signalling pathways [28–30], contributing to metabolic reprogramming of cancer cells [31,32].

### 3.1. CircRNA and cancer glucose metabolism

Normal cells consume glucose by oxidative phosphorylation under aerobic conditions to generate ATP. While glycolysis occurs only under hypoxia, glycolysis is the common way for cancer cells to utilize sugar, even in an oxygen-rich environment, which is known as the Warburg Effect. Most circRNAs play a carcinogenic role by regulating cancer glucose metabolism [33,34], and only a small part of circRNAs inhibits the occurrence and development of cancer through glucose metabolism [35–38] (Table 1). To complete glycolysis in cancer cells, glucose transporter (GLUT) and such enzymes as hexokinase (HK), phosphofructokinase (PFK), pyruvate kinase (PK), lactate dehydrogenase A (LDHA), enolase 1 (Recombinant), Human Enolase-1, ENO1, pyruvate dehydrogenase kinases (PDKs) work jointly to meet the enormous demand for energy.

**Table 1.** CircRNA and cancer glucose metabolism.

CircRNA	Cancer type	Expression	Mechanism	Ref.
circDENND4C	colorectal cancer	up	circDENND4C/miR-760/GLUT1	[28]
circMYLK	non-small cell lung cancer	up	circMYLK/miR-195-5p/GLUT3	[39]
circCDKN2B-AS1	cervical cancer	up	circCDKN2B-AS1/IMP3/HK2	[27]
circUBE2Q2	gastric cancer	up	circUBE2Q2/miR-370-3p/STAT3/PFK/HK2	[41]
circVAMP3	renal cell carcinoma	up	circVAMP3/FGFR1/LDHA	[43]
circ-ENO1	lung adenocarcinoma	up	circ-ENO1/miR-22-3p/ENO1	[24]
circ_0002711	ovarian cancer	up	circ_0002711/miR-1244/ROCK1/ PDK1	[44]
ciRS-122	colorectal cancer	up	ciRS-122/miR-122-PKM2	[40]
circEPHB4	gliomas	up	circEPHB4/miR-637/PDK1	[45]
circ0091579	hepatocellular carcinoma	up	circ0091579/miR-1287/PDK2	[46]
circCCDC66	thyroid cancer	up	circCCDC66/miR-211-5p/PDK4	[47]
circRNF20	breast cancer	up	circRNF20/miR-487a/HIF-1α	[26]
circECE1	osteosarcoma	up	circECE1/c-Myc/TXNIP	[31]
circ-ACACA	lung cancer	up	circ-ACACA/miR-1183/c-Myc	[50]
circCUX1	neuroblastoma	up	circCUX1/EWSR1/MAZ	[51]
circNRIP1	gastric cancer	up	circNRIP1/miR-149-5p/AKT1/mTOR	[52]
circ_100395	papillary thyroid cancer	down	circ_100395/PI3K/AKT/mTOR	[53]
circ_0001610	endometrial cancer	up	circ_0001610/miR-646/STAT3	[54]
circAKT3	lung cancer	up	circAKT3/miR-516b-5p/STAT3	[55]
circ-CSNK1G1	triple-negative breast cancer	up	circ-CSNK1G1/miR-28-5p/LDHA	[42]
circ_0006089	gastric cancer	up	circ_0006089/miR-361-3p/TGFB1	[34]
circ_0004872	oral squamous cell carcinoma	down	circ_0004872/miR-424-5p	[35]
circ_0003215	colorectal cancer	down	circ_0003215/miR-663b/DLG4/G6PD	[36]
circ_0094343	colorectal cancer	down	circ_0094343/miR-766-5p/TRIM67	[37]
circCDK17	cervical cancer	up	circCDK17/miR-1294/YWHAZ	[33]
circ_0086414	esophageal cancer	down	circ_0086414/miR-1290/SPARCL1	[49]
circHEATR5B	glioblastoma multiforme	down	ZCRB1/circHEATR5B/HEATR5B-881aa/JMJD5/PKM2	[38]
circDNMT1	gastric cancer	up	circDNMT1/miR-576-3p/HiF-1	[48]
circPUM1	esophageal squamous cell carcinoma	up	___	[57]
circNFATC3	Breast cancer ovarian cancer	up	___	[56]

#### 3.1.1. CircRNAs regulate glucose transporters

The main role of GLUT is to transport extracellular glucose into the cell [30]. CircDENND4C and GLUT1 are found to be upregulated in colorectal cancer tissues and cell lines. Glycolysis in colorectal cancer cells was inhibited after knocking down circDENND4C and GLUT1; and as a result, cell proliferation and migration were significantly reduced. miR-760 which can bind to GLUT1 and inhibit its expression, is the direct target of circDENND4C; thus, circDENND4C upregulates GLUT1 by adsorbing miR-760, promoting cell glycolysis, increasing lactate level, and promoting the proliferation and migration of colorectal cancer cells [28]. Xiong [39] reported that circMYLK is significantly upregulated in non-small cell lung cancer tissues and cell lines, and its high expression is closely related to the malignant features of the tumour and poor prognosis of patients. CircMYLK is the molecular sponge of miR-195-5p whose target gene is GLUT3. When GLUT3 is inhibited, the efficiency of aerobic glycolysis in cancer cells is reduced, resulting in the decreased production of lactic acid. Overexpressed circMYLK promotes GLUT3 expression via adsorbing miR-195-5p, promoting cancer cell glycolysis as well as the malignant phenotype.

#### 3.1.2. CircRNAs regulate enzymes

**Figure 1.**
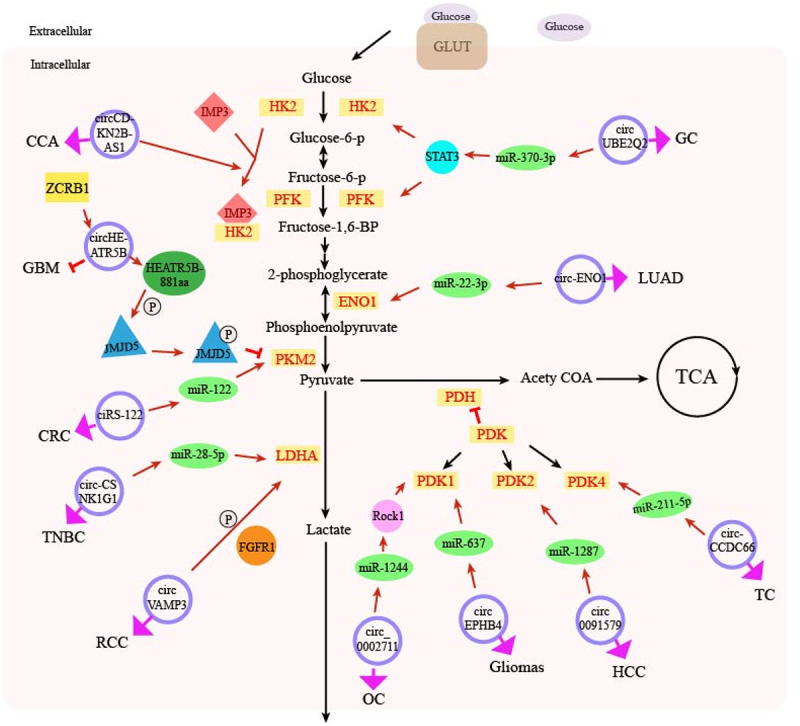
Schematic diagram of the effect of circRNA regulation of glucose metabolism-related enzymes on cancer glucose metabolism. It shows how circRNAs can affect the expression of downstream target genes by antagonizing miRNA or interacting with RBP, thereby increasing or decreasing the level of glucose metabolism related enzymes. The whole process promotes or inhibits cancer progression. The blue circle represents various circRNAs, the green ellipse represents miRNA, and the light yellow rectangle represents glucose metabolism related enzymes. The positive relationship is indicated by arrows (black arrow: glucose metabolism pathway, red arrow: pathway of circRNA function, pink arrow, and the role of circRNA in related cancers), and the negative relationship is indicated by a short line. P on the small white circle represents phosphorylation. GC, gastric cancer; LUAD, lung adenocarcinoma; TC, thyroid cancer; HCC, hepatocellular carcinoma; OC, ovarian cancer; RCC, renal cell carcinoma, TNBC, triple negative breast cancer; CRC, colorectal cancer; GBM, glioblastoma multiforme; CCA, cervical cancer. HK2, hexokinase 2; PFK, phosphofructose kinase; EON1, enolase; 1; PKM2, pyruvate kinase M2; LDHA, lactic dehydrogenase; PDH, pyruvate dehydrogenase; PDK, pyruvate dehydrogenase kinase; TCA, tricarboxylic acid cycle, Acety COA, acetyl coenzyme A.

CircRNAs affect cancer progression by regulating glucose metabolism-related enzymes (Figure 1). After glucose enters the cell, three types of rate-limiting enzymes – HK, PFK and PK function in sequence. HK converts glucose into glucose 6-phosphate, PFK catalyses fructose 6-phosphate to fructose 1,6-diphosphate, and PK makes phosphoenolpyruvate to pyruvic acid. M2 type of PK (PKM2) that expresses highly in drug-resistant colorectal cancer cells is an isomer of PK. Oxaliplatin-resistant colorectal cancer cells transport ciRS-122 to oxaliplatin-sensitive cancer cells via exosomes, and ciRS-122 adsorbs miR-122 in drug-sensitive cancer cells, upregulating PKM2, promoting glycolysis of cells and reducing cell susceptibility to drugs [40]. CircCDKN2B-AS1 is upregulated in cervical cancer and precancerous tissues and is positively correlated with HK2 mRNA expression [27]. Insulin-like growth factor mRNA binding protein 3 (IMP3) is an RBP that regulates glycolysis levels in cancer cells and binds to 3’UTR of HK2 mRNA, which can be weakened by knocking down circCDKN2B-AS1; therefore, the stability of HK2 mRNA would be destroyed, the expression of HK2 declines and glycolysis in cervical cancer cells is inhibited [27]. In gastric cancer tissues, the expression of HK2 and PFK is decreased when STAT3 is knocked down. CircUBE2Q2 acts as a sponge for miR-370-3p, activating the STAT3 pathway to promote PFK and HK2 expression, thereby increasing glycolysis levels in gastric cancer cells and promoting cell proliferation and migration [41].

**Figure 2.**
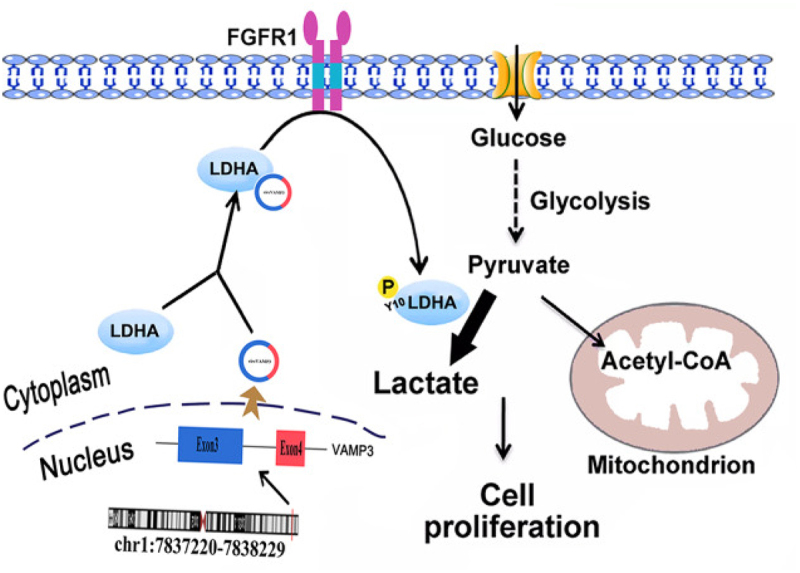
Schematic diagram of circVAMP3 faciliats renal cell carcinoma progression. CircVAMP3 is formed by reverse splicing of VAMP3. It directly binds to LDHA and phosphorylates Y10 of LDHA through upstream kinase FGFR1 to activate LDHA. LDHA acts on pyruvate to convert it into lactic acid. This process promotes the proliferation of renal cell carcinoma cells [43]. Copyright 2022 Springer Nature.

LDHA performs the final step of aerobic glycolysis, catalysing pyruvate into lactic acid. CircRNA affects cancer glucose metabolism by regulating LDHA [42]. In human renal cell carcinoma, circVAMP3 expression is significantly upregulated and positively correlated with TNM stage [43]. Besides, circVAMP3 is the first circRNA found to interact directly with LDHA, which promotes phosphorylation at the Y10 site of LDHA via fibroblast growth factor receptor 1 (FGFR1), enhancing the activity of LDHA, increasing lactate production, and promoting cancer cell proliferation (Figure 2). Finally, monocarboxylic acid transporters transport lactate into the extracellular matrix. The mechanism by which circRNA regulates monocarboxylic acid transporters and subsequently influences cancer progression has not yet been studied.

CircRNA also regulates other enzymes to influence the development of malignant tumours besides the glycolytic rate-limiting enzymes HK2 and PKM2. ENO1 plays the key role in aerobic glycolysis as a glycolytic enzyme by converting 2-phosphoglycerol into Phosphoenolpyruvate. Circ-ENO1 upregulates the expression of ENO1 by adsorbing miR-22-3p, thus, promotes glycolysis of lung adenocarcinoma cells and tumour progression [24]. The study also reveals that circ-ENO1 promotes glycolysis, proliferation, and EMT in lung adenocarcinoma by upregulating its host gene ENO1 [24].

The pyruvate dehydrogenase complex (PDHC) is regulated by PDK and is essential in sugar metabolism by converting pyruvate into acetyl-CoA entering the citric acid cycle. PDK inhibits activity of PDHC by phosphorylating its specific serine residues. Rho-associated coiled-coil forming protein kinase 1 (ROCK1) can be combined with activated Rho protein in tumours, promoting the cell actin backbone recombination in cancer, thus, accelerating the cancer cell proliferation and migration. Xie [44] and the colleagues knocked out circ_0002711 in ovarian cancer cells and found that by modulating the miR-1244/ROCK1 axis, glucose consumption, lactate production, and the expression of PDK1, one of the subtype of PDK, were significantly inhibited; thereby, the cell proliferation and migration was inhibited. In gliomas, circRNA EPHB4 adsorbs miR-637, promotes lactic acid production and PDK1 expression [45]. In HCC, circ0091579 binds to miR-1287 under hypoxic stress to enhance the protein expression of PDK2, the target gene of miR-1287, promotes glucose uptake and lactate production, and exerts pro-cancer effects [46]. In addition, circCCDC66 in thyroid cancer acts as a sponge for miR-211-5p, which enhances the expression of PDK4 protein, promotes the proliferation, migration, and invasion of thyroid cancer [47].

#### 3.1.3. CircRNAs regulate transcription factors

Many circRNAs affect glycolysis by modulating transcription factors [48,49]. Hypoxia inducible factor-1α (HIF-1α) is a nuclear transcription factor produced by cancer cells adapting to hypoxia environments. HIF-1α can bind to the promoter of HK2, facilitate the HK2 transcription, and the glycolysis [26]. CircRNF20 is reported to bind to miR-487a, upregulating HIF-1α to accelerate glucose uptake and lactic acid production.

MYC, an oncogene, is often regulated abnormally in tumorigenesis. C-Myc, a transcription factor encoded by MYC, could regulate the expression of TXNIP which is well known as the negative regulator of glucose metabolism. Chen [31] has proved that circECE1 is mainly located in the cytoplasm and highly expressed in osteosarcoma when compared with the normal cells. When circECE1 had been knocked out, TXNIP was significantly increased on both mRNA and protein level; therefore, TXNIP transcription was inhibited in osteosarcoma, and subsequently activated the Warburg Effect [31]. CircECE1 interacts with c-Myc to protect it from ubiquitination and degradation, activates c-Myc-TXNIP signalling pathway, boosts glucose uptake, glycolysis, lactate production, and ATP production to facilitate the proliferation and migration of osteosarcoma [31]. Circ-ACACA increases the expression of c-Myc, MMP9, and GLUT1 in lung cancer by interacting with miR-1183, motivating glucose intake, glycolysis, and accelerating the cell proliferation [50]. Cut-like homeobox 1 (CUX1), the transcription factor involved in embryonic development, could regulate the cancer cell proliferation, migration, and EMT. Studies [51] have proved that circCUX1 is produced by CUX1, binds to the Ewing sarcoma breakpoint region 1 gene (EWSR1), facilitates its interaction with MAZ, the MYC-associated zinc finger protein, and activates MAZ. Finally, it is the MAZ trans-activation that affects the transcription of CUX1 as well as the tumour progression. On the other side, CUX1 could increase the expression of ENO1, glucose-6-phosphate isomerase (GPI); followed by glucose-6-phosphate converting to fructose-6-phosphate during glycolysis [51]. Thus, overexpressed circCUX1 accelerates the glucose uptake, lactic acid production, and ATP provision via the circCUX1/EWSR1/MAZ axis. In neuroblastoma, knocking out circCUX1 significantly inhibits the glycolysis, and the results were that the tumour growth, invasion, migration and metastasis were frustrated [51].

#### 3.1.4. CircRNAs regulate signalling pathways

CircRNA can also influence cancer cell glucose metabolism by modulating many signalling pathways. The phosphoinositide-3-kinase (PI3K)/AKT/mammalian target of Rapamycin (mTOR) axis is the classic signalling pathway that maintains energy homoeostasis via energy producing activities. AKT phosphorylates the T246 locus of mTORC1, resulting in mTORC1 activation. Zhang [52] found that circNRIP1 was significantly highly expressed in human gastric cancer. Overexpressed circNRIP1 increased its adsorbing miR-149-5p; then the expression of AKT1 and mTOR which is the downstream gene of AKT1 were increased, boosting the production of lactic acid and ATP, and promoting the development of gastric cancer [52]. Study [53] have found that circ_100395 inhibits glucose uptake and lactic acid production in papillary thyroid cancer by suppressing the PI3K/AKT/mTOR signalling pathway, and the levels of glycolysis, thus the cell invasion and migration are reduced.

The glucose metabolism in cancer cells is also regulated by Signal Transducer and Activator of Transcription (STAT) signalling pathways, which affects the proliferation and metastasis of cancer. STAT3, one of the most promising new anti-cancer targets in STAT family, is the potential target of miR-646. Circ_0001610 is highly expressed in endometrial cancer and can upregulate the level of STAT3 by adsorbing miR-646; thereby, upregulating the level of HK2 and LDHA and promoting cellular glycolysis [54]. CircAKT3 and STAT3 were highly expressed in human lung cancer, and miR-516b-5p were simultaneously downregulated. The sensitivity to cisplatin in lung cancer was increased after circAKT3 had been knocked down; and therefore, the glucose consumption as well as the lactic acid formation were inhibited via the miR-516b-5p/STAT3 axis, finally, HIF-1α-dependent glycolysis was hindered [55].

However, in certain cancers, not only does the glycolysis increase, but the oxidative phosphorylation upregulates [56,57]. Experimental data suggest that the level of oxidative phosphorylation is also upregulated when circRNAs affect increased levels of glycolysis in cancer cells [56,57]. CircPUM1 [57] has been reported to have the tumorigenic effects on human oesophageal squamous cell carcinoma. Interestingly, neither being knocked down nor overexpressed did circPUM1 affect cell glycolysis; whereas, oxygen consumption, basal respiration, and maximum respiration increased significantly after circPUM1 was overexpressed. The proteins of mitochondrial complex III participate in the oxidative respiration chain. CircPUM1 interacted with the core protein II of the ubiquinone cytochrome C reductase and also acted as the scaffold proteins of mitochondrial complex III to promote oxidative phosphorylation in cancer cells [57]. In the hypoxic microenvironment, the accumulation of HIF1-α promotes the glycolysis, maintaining the survival of the cancer cells. The expression level of circPUM1 showed a negative correlation with the degradation of HIF1-α. HIF1-α might be involved in regulating the glycolysis and the oxidative phosphorylation in human oesophageal squamous cell carcinoma, the underlying mechanism has yet to be studied [57]. Study [56] have found that circNFATC3 is highly expressed in breast and ovarian cancer, and silenced circNFATC3 slows cancer cell proliferation, migration, and invasion. When circNFATC3 had been knocked down, both the oxygen consumption rate (OCR) and extracellular acidification rate (ECAR) were all decreased, which represented the ability of intracellular oxidative phosphorylation and glycolysis, respectively; showing that circNFATC3 could facilitate the oxidative phosphorylation and glycolysis in cancer cells; further, which genes and pathways worked in these regulating activity remains to be found [56].

### 3.2. CircRNA and lipid metabolism in cancer

Accelerated lipid production is another feature of cancer cell metabolism. To maintain the integrity of the cell membranes, cancer cells need unsaturated fatty acids to synthesize their cell membranes; cancer cells, on the other hand, acquire endogenous fatty acids through lipolysis and carry out β-oxidation to provide energy for their proliferation. It has been found that circRNAs can promote cancer proliferation, migration and invasion by affecting lipid metabolism in malignant tumours [58] (Table 2). CircACC1 forms the ternary complex with the β1 and γ1 subunits of AMP-activated protein kinase (AMPK) to stabilize and promote the enzymatic activity of AMPK as a whole enzyme [59]. AMPK whose active form facilitates the fatty acid oxidation plays a key role in maintaining cellular energy homoeostasis. In human colorectal cancer, circACC1 reduces the concentration of free fatty acids via enabling the assembly and activation of AMPK. When circACC1 was knocked down, the β-oxidation of the cancer cells, the proliferation, migration, and invasion of cancer were all depressed [59].

**Table 2.** CircRNA and lipid metabolism in cancer.

CircRNA	Cancer type	Expression	Mechanism	Ref.
circACC1	colorectal cancer	up	circACC1/AMPK	[59]
circCUX1	neuroblastoma	up	circCUX1/p113/ALDH3A1/ZRF1/BRD4	[25]
circPTK2	cachexia	up	circPTK2/miR-182-5p/JAZF1	[60]
circRIC8B	chronic lymphocytic leukaemia	up	circRIC8B/miR-199b-5p/LPL	[58]

**Figure 3.**
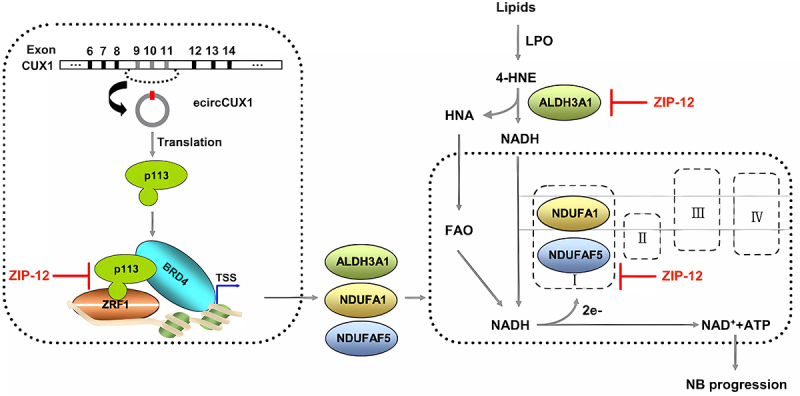
The mechanism of ecircCUX1 encodes P311 protein. p113, together with ZRF1 and BRD4, activates the transcription of NDUFAF5, NDUFA1 or ALDH3A1 to promote the conversion of fatty aldehyde to fatty acid and the β-oxidation, enhance the activity of mitochondrial complex I. This process promotes the occurrence and development of neuroblastoma. In addition, ZIP-12 blocks the p113-ZRF1 interaction and inhibits the progression of neuroblastoma [25]. Copyright 2021 Springer Nature.

In neuroblastoma, the expression of p113, encoded by exonic circCUX1, was higher than that of the normal ganglia, and was negative correlation with the patient overall survival [25]. P113 composed the ternary complex with ZRF1 and BRD4 (transcription factors), activates A1 which is the member of the aldehyde dehydrogenase-3 family, converts 4-hydroxynenoic acid (lipid peroxidation product) into fatty acids; subsequently, enormous fatty acids proceed β-oxidize, providing adequate energy for the proliferation, migration and invasion of neuroblastoma [25] (Figure 3).

Cachexia is a destructive pathological disease, which is common in advanced stages of cancer. Its main characteristics are skeletal muscle atrophy and fat storage depletion. Study [60] has shown that circPTK2 is upregulated in the adipose tissue of the cancer patients with cachexia. The lipolysis marker – fat triglyceride lipase and hormone-sensitive lipase were downregulated after circPTK2 had been knocked down; on the other side, the expression of such lipogenesis markers as fatty acid-binding protein 4 was increased [60]. Overexpression of the juxtaposed another zinc finger gene (JAZF1) in fat cells and the liver cells reduces lipid synthesis and increases the lipolysis. CircPTK2 adsorbs miR-182-5p competitively, thus, blocks the inhibitory effect of miR-182-5p on JAZF1, increases the lipolysis markers, and reduces lipogenesis markers which has mentioned above [60]. The lipid droplets became less significantly showed by oil red staining. The research on this pathway will be promising as for improving the malignant adipose exhaustion in the patients with cancer cachexia.

### 3.3. CircRNA and amino acid metabolism in cancer

Take glutamine for example: its metabolism features in malignant tumour was observed in the human cervical cancer cell line – Hela cells in 1950. Compared to other kind of amino acids, Harry Eagle discovered that only by acquiring excess glutamine could Hela cells achieve the optimal growth [3]. In addition, cancer tissues consumed glutamine with much faster speed than the normal tissue surrounding them. Glutamine produced by cancer cells themselves cannot meet the needs of their rapid proliferation, more glutamine intake must be fulfilled from extracellular environments through membrane transporters; the other way was to enhance the expression and activity of key metabolic enzymes in the glutamine metabolic pathway to meet the needs of cell proliferation [61–63]. CircRNAs affect cancer amino acid metabolism by regulating glutamine metabolism and serine metabolism (Table 3).

**Table 3.** CircRNA and amino acid metabolism in cancer.

CircRNA	Cancer type	Expression	Mechanism	Ref.
circ-MBOAT2	pancreatic cancer	up	circ-MBOAT2/miR-433-3p/GOT1	[61]
circ-CREBBP	glioma	up	circ-CREBBP/miR-375/GLS	[64]
circ_0000003	tongue squamous cell carcinoma	up	circ_0000003/miR-330-3p/GLS	[65]
circRUNX1	colorectal cancer	up	circRUNX1/miR-485-5p/SLC38A1	[66]
circ_0001273	esophageal cancer	up	circ_0001273/miR-622/SLC1A5	[62]
circ_0000463	non-small-cell lung cancer	up	circ_0000463/miR-924/SLC1A5	[63]
circAKT3	gastric cancer	up	circAKT3/miR-515-5p/SLC1A5	[70]
circ-SFMBT2	esophageal cancer	up	circ-SFMBT2/miR-107/SLC1A5	[71]
circSEPT9	breast cancer	up	circSEPT9/miR-149-5p/SLC1A5	[72]
circ_0000518	non-small-cell lung cancer	up	circ_0000518/miR-330-3p/SLC1A5	[68]
circ_0018189	non-small-cell lung cancer	up	circ_0018189/miR-656-3p/SLC7A11	[73]
circ_0000808	non-small-cell lung cancer	up	circ_0000808/miR-1827/SLC1A5	[67]
circ_0062558	triple-negative breast cancer	up	circ_0062558/miR-876-3p/SLC1A5	[29]
circ_0025033	ovarian cancer	up	circ_0025033/miR-370-3p/SLC1A5	[69]
circ_0000517	non-small cell lung cancer	up	circ_0000517/miR-330-5p/YY1	[74]
circHMGCS1	hepatoblastoma	up	circHMGCS1/miR-503-5p/IGF2/IGF1R	[32]
circMYH9	colorectal cancer	up	circMYH9/hnRNPA2B1/p53 pre-mRNA	[75]

#### 3.3.1. CircRNAs regulate enzymes

In pancreatic cancer, circ-MBOAT2 was upregulated to promote the glutamic acid production as well as the glutamine utilization. While miR-433-3p expression was upregulated, the converse results came out [61]. The inhibitory effect of miR-433-3p on glutamine production and consumption can be reversed by the overexpression of Glutamate Oxaloacetate Transaminase 1 (GOT1) which promotes glutamine metabolism. CircRNA also stimulates glutamine metabolism by regulating glutaminase (GLS) who catalysed the hydrolysis of L-β-glutamine to L-glutamic acid and ammonia. Circ-CREBBP was reported to be upregulated in gliomas, accelerating the cell proliferation [64]. Circ-CREBBP regulated the expression of GLS by adsorbing miR-375, accelerating the glutamine metabolism, thus boosting the glioma progress. In the tongue squamous cell carcinoma [64], Circ_0000003 regulated the GLS expression and increased glutamine catabolism via adsorbing miR-330-3p, promoting the cell proliferation, migration, and invasion [65].

#### 3.3.2. CircRNAs regulate transporters

**Figure 4.**
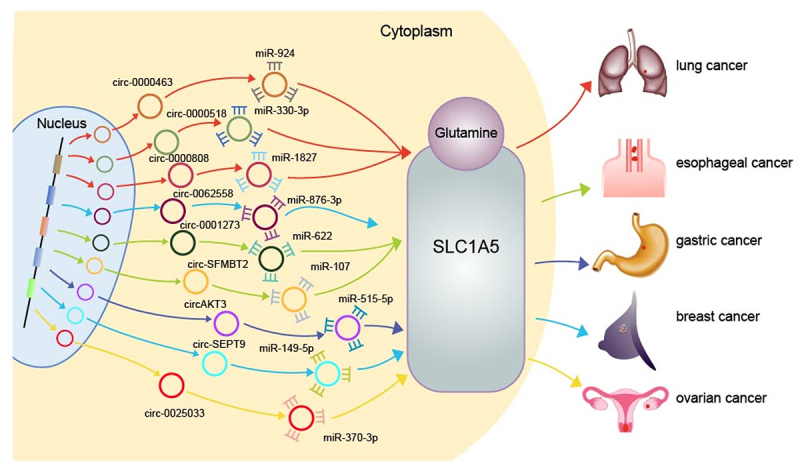
Schematic diagram of circRNAs affecting cancer amino acid metabolism through glutamine transporter SLC1A5. It shows how circRNAs can promote cancer amino acid metabolism by adsorbing miRNA to regulate SLC1A5. The circle represents circRNAs; the shapes of teeth represent miRNAs; a positive relationship is indicated by an arrow. (red represents promoting lung cancer; green represents promoting oesophageal cancer; dark blue represents promoting gastric cancer; light blue represents promoting breast cancer; yellow represents promoting ovarian cancer).

When circRUNX1 was knocked down, the proliferation, migration, invasion as well as the glutamine decomposition were inhibited in colorectal cancer cells; and the cell apoptosis was induced [66]. The tumour growth was blocked *in vivo*. The mechanism was that CircRUNX1 directly bound to miR-485-5p, thus raised the concentration of glutamine via upregulating the expression of SLC38A1 in cancer cells [66]. SLC38A1 and SLC1A5 are essential members of the solute carrier (SLC) family which transport glutamine into the cells from the outside. SLC1A5 is upregulated in variety of cancers, promoting glutamine metabolism. It has been found that multiple circRNAs can regulate SLC1A5 through their miRNA sponge role; therefore, the amino acid metabolism could be affected in non-small cell lung cancer [63,67,68], ovarian cancer [69], oesophageal cancer [62] and other cancer cells [70–72] (Figure 4); and as the results, the development of malignant tumours would be promoted or inhibited. It has been found that circRNA can also promote the occurrence and development of non-small cell lung cancer by up regulating SLC7A11 of SLC family [73].

#### 3.3.3. CircRNAs regulate transcription factors and signalling pathways

**Figure 5.**
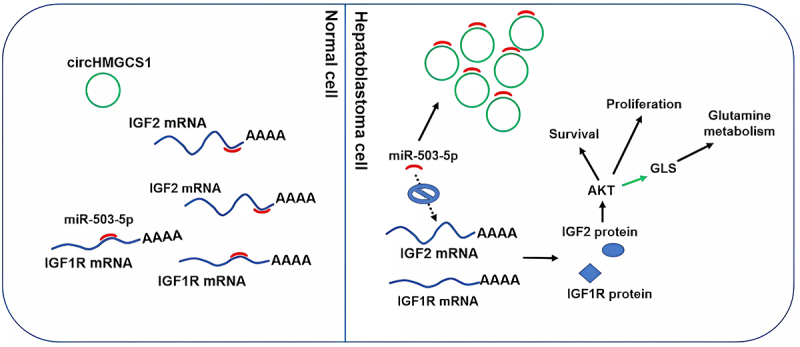
Molecular mechanism of circHMGCS1 promoting hepatoblastoma progression: The left side of the picture shows the distribution of several substances in normal cells. The picture on the right shows that in hepatoblastoma cells, circHMGCS1 upregulates the target gene IGF by adsorbing miR-503-5p. The increase of IGF protein activates AKT signal pathway to promote cancer cell proliferation and shorten the survival period of patients with hepatoblastoma. In addition, it also promotes amino acid metabolism in cancer cells by increasing glutaminase [32]. Copyright 2019 Ivyspring International Publisher.

Zhong [74] found that after circ_0000517 was silenced, the glutamine consumed in non-small cell lung cancer cells was reduced compared with that in the control group; besides, the glutamic acid content and α-ketoglutaric acid, the downstream key metabolite of glutamate, were also significantly reduced. Circ_0000517 regulated YY1 (the transcription factor) via adsorbing miR-330-5p, stimulating glutamine consumption; thus the glutamic acid content and α-ketoglutaric acid increased, and cell proliferation was accelerated [74]. Zhen [32] found that in hepatoblastoma, circHMGCS1 and insulin-like growth factor 2 (IGF2) were significantly upregulated and negatively correlated with the prognosis of patients, which is expected to be the biomarkers for the diagnosis and prognosis. Further studies showed that circHMGCS1 is the sponge of miR-503-5p which is involved in regulating the PI3K-Akt signal path, the important downstream pathway of IGF2/IGF1R [32] (Figure 5). The knocked-down circHMGCS1 significantly inhibited the activation of Akt. Whether Akt is activated or not reflects the effect of the circHMGCS1/miR-503-5p axis on the expression of IGF2/IGF1R [32]. The PI3K-Akt pathway has been reported to regulate the glutamine utilization intracellularly by stimulating glutamine uptake, GLS, and glutamine synthase. Except for the glutamine metabolism, circRNA could also promote the amino acid metabolism process of cancer through serine metabolism. HnRNPA2B1 which binds to nascent RNA is a reader of nuclear N6-methyladenosine (m6A), thus affects a range of complicated metabolism. P53 inhibits the expression of phosphoglycerol dehydrogenase which is the rate-limiting enzyme of the serine biosynthetic pathway; therefore, the expression of serine is decreased. HnRNPA2B1 can guarantee the stability of p53 through the interaction with p53 pre-mRNA. Whereas, circMYH9 inhibits the stability of p53 pre-mRNA by blocking hnRNPA2B1 from binding to p53 pre-mRNA, increasing endogenous serine production [75].

### 3.4. CircRNA and exosomal

So far, few exosomal circRNAs have been found to be involved in the metabolic reprogramming of cancer cells. For example, four exosomal circRNAs, circPDK1 [76], circ-RNF121 [77], circ_0072083 [78], and circFNDC3B [79] promote glycolysis in cancer cells by acting as molecular sponges for miRNAs and thus contribute to cancer progression. Yang found that exosomes-derived hsa_circ_0085361 promoted glutamine metabolism in bladder cancer cells by up-regulating the expression of GLS by adsorbing miR-141-3p [80]. In summary, exosomal circRNAs play an important role in tumour metabolic reprogramming, and exosomal circRNA is beneficial to the non-invasive diagnosis of cancer in clinical practice. Therefore, the study of exosomal circRNA is a trend in the future.

## 4. Conclusion and perspectives

With the continued examination of circRNA, it has been elucidated that circRNA has stability, universality and diversity, and it can be packaged by exosomes and transported to the peripheral blood. These characteristics make circRNA a potentially substantial biomarker. Secondly, energy metabolism is a recent research hotspot and this article focuses on the review of many circRNAs involved in multi-level, multi-substance metabolic reprogramming of malignant tumours. CircRNA affects the occurrence and development of cancer mainly by regulating the transport proteins, enzymes, signal pathways and related transcription factors of key substances in the carbohydrate metabolism and amino acid metabolism of cancer cells, while there is less research on lipid metabolism. CircRNA affects the activity of cancer cells through enzymes in the metabolic process. CircRNA plays a role mainly through the adsorption of miRNA and the binding of RBP in the three kinds of metabolism of cancer cells. Oxidative phosphorylation is an indispensable part of the whole energy metabolism process, but the specific mechanism of circRNA’s role in oxidative phosphorylation in cancer cells remains to be explored.

In addition, in recent years, studies have been conducted on the regulation of cancer metabolic reprogramming by circRNA, but few of them have been clinically applied as a diagnostic cancer biomarker, which may be due to the lack of clinical patient tissues and blood to verify the clinical applicability of circRNA. In addition, circRNA research has the shortcomings of complex experimental means and detection difficulty. To sum up, the availability of clinical samples of circRNA for research needs to be improved as well as developing faster and less costly circRNA detection.

In conclusion, clarifying the characteristics and mechanisms of metabolic disorder in malignant tumours may provide new possibilities for tackling cancer. Moreover, on this basis, continued exploration of methods of targeting circRNA and intervention in the energy supply chain of malignant tumours could provide new ideas for the development of clinical drugs as well as detection and monitoring programmes.
